# An Unusual Case of Acquired Angioedema and Monoclonal Gammopathy of Renal Significance in a Middle-Aged Caucasian Female

**DOI:** 10.1177/2324709620912096

**Published:** 2020-03-13

**Authors:** Sasmit Roy, Venu Madhav Konala, Thurein Kyaw, Sandipan Chakraborty, Srikanth Naramala, Vijay Gayam, Sreedhar Adapa, Subhasish Bose

**Affiliations:** 1 Lynchburg Nephrology Physicians, Lynchburg, VA, USA; 2 Ashland Bellefonte Cancer Center, Ashland, KY, USA; 3 Miami Valley Hospital, Dayton, OH, USA; 4 Adventist Medical Center, Hanford, CA, USA; 5 Interfaith Medical Center, Brooklyn, NY, USA; 6 The Nephrology Group, Fresno, CA, USA

**Keywords:** acquired C1 esterase inhibitor deficiency, acquired angioedema, acute kidney injury, monoclonal gammopathy of renal significance, proliferative glomerulonephritis with monoclonal immunoglobulin deposition

## Abstract

Acquired angioedema due to deficiency of C1 esterase inhibitor is also called acquired angioedema and is abbreviated as C1INH-AAE. It is a rare syndrome of recurrent episodes of angioedema, without urticaria, and in some patients, it is associated with B-cell lymphoproliferative disorders. Kidney involvement is rare in this condition. The monoclonal immunoglobulin secreted by a nonmalignant or premalignant B-cell or plasma cell clone, causing renal damage that represents a group of disorders which are termed as monoclonal gammopathy of renal significance (MGRS). In this article, we report a rare case of acquired C1 esterase deficiency angioedema and acute kidney injury with renal biopsy-proven MGRS. We present a 64-year-old Caucasian woman who presented with 2 weeks of recurring urticaria and new onset of acute kidney injury. She was diagnosed with monoclonal gammopathy–associated proliferative glomerulopathy through kidney biopsy, and serological workup came back positive for C1 esterase deficiency, implying acquired angioedema. Acquired angioedema is a rare disease with systemic involvement. Recurrent allergic manifestations and acute kidney injury should prompt MGRS as a differential.

## Introduction

The monoclonal immunoglobulin (MIg) secreted by a nonmalignant or premalignant B-cell or plasma cell clone causes renal damage that represents a group of disorders that are termed as monoclonal gammopathy of renal significance (MGRS). The renal injury is caused by direct deposition or indirect functional interference of MIg.^1-3^ The hematological abnormality in patients with MGRS is generally consistent with monoclonal gammopathy of undetermined significance (MGUS) and does not meet the criteria for symptomatic multiple myeloma or lymphoma. The renal prognosis of MGRS is not benign. MGRS can cause multiple renal manifestations of which cast nephropathy, MIg deposition disease, and renal amyloidosis are more predominant. Proliferative glomerulonephritis with MIg deposition (PGNMID) is a monoclonal gammopathy–associated kidney disease that mimics immune-complex glomerulonephritis.^4,5^ Patients can present with renal insufficiency, hematuria, and nephrotic syndrome.^4^

Acquired C1 esterase inhibitor deficiency is an infrequent condition that is often related to underlying lymphoproliferative disorders and autoimmune diseases. It usually presents after the second decade of life. This contrasts with hereditary C1 esterase inhibitor deficiency, which is inherited as an autosomal dominant trait and presents earlier in life.^6^ The most common clinical presentation is painless swellings subsiding over a few days, which is similar in both forms.

A similar clinical presentation with low C1 esterase inhibitor concentration in elderly patients should raise suspicion for B-cell neoplasm. Multiple myeloma can sometimes present in this manner.^7^ In this article, we present a patient whose paraprotein was discovered on renal biopsy, and subsequently, further investigations showed spurious low C1q, C3, and C4 complement level.

## Case Presentation

A 64-year-old Caucasian female presented to the emergency department with complaints of waxing and waning maculopapular rashes in both upper and lower extremities along with chills accompanied by lower extremity swelling.

On examination, she was alert, awake, and could breathe normally and swallow but had hoarseness of voice. She also had 2+ pedal edema in both lower extremities. She had bilateral nonblanching papular rashes in both lower extremities. Her blood pressure and heart rate were essentially normal, and she was saturating 98% on room air. The patient denied any family history of angioedema. She had a past medical history of essential hypertension, on and off urticaria with lip swelling, and paroxysmal tachycardia. She was not taking an angiotensin-converting enzyme inhibitor. She was getting short courses of 3 to 5 days of oral prednisone at urgent care. Her rash subsided with steroids but recurred on completion of steroids.

Routine blood samples taken on admission showed an increase in urea and creatinine at 44 mg/dL (reference range = 5-23) and 2.4 mg/dL (reference range = 0.5-1.3), respectively. The serum calcium level was normal. Erythrocyte sedimentation rate was increased at 63 mm/h (reference range <20). All other routine biochemistry was unremarkable. A full blood count showed mild leukocytosis (white blood cells count = 11500/µL, reference range <10 000/µL). She had no anemia. Urine analysis showed 1+ proteinuria and 1+ hematuria. The urine protein creatinine ratio was 0.19. There were no casts visible on the urinary microscope examination.

Complement levels, hepatitis panel, vasculitis workup, and immunoglobulin studies were requested as part of a standard protocol to investigate glomerulonephritis and angioedema. Hepatitis B and C, antinuclear antibody, double-stranded deoxyribonucleic acid, anti-Sjogren’s syndrome A and B, anti-neutrophil cytoplasmic antibody, and cryoglobulins were all negative.

The C4 concentration was extremely low at <2 mg/dL (reference range = 14-44), C3 was very low at 23 mg/dL (reference range = 92-190), and C1 q level was low at 1.3 mg/dL (reference range = 11.8-24.4). C1 esterase inhibitor level came high at 48 mg/dL (reference range = 21-39).

**Figure 1.**
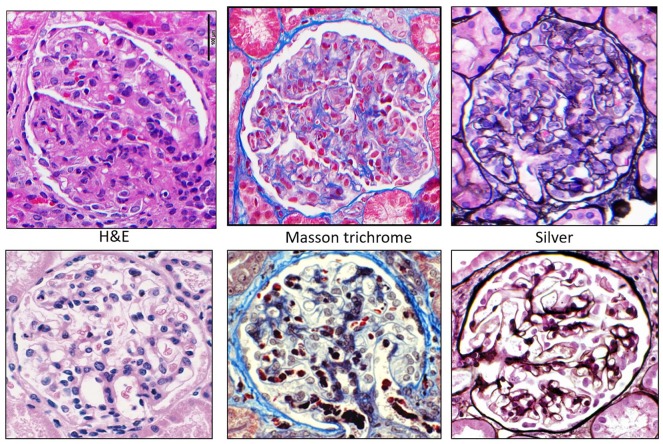
Glomeruli with global endocapillary hypercellularity with leukocytes (mainly monocytes/macrophages) obliterating most capillary lumens (top) compared with normal (bottom).

**Figure 2.**
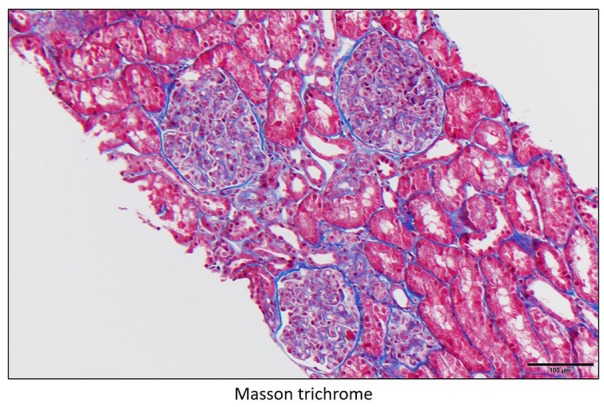
Glomeruli with global endocapillary hypercellularity with leukocytes (mainly monocytes/macrophages) obliterating most capillary lumens.

**Figure 3.**
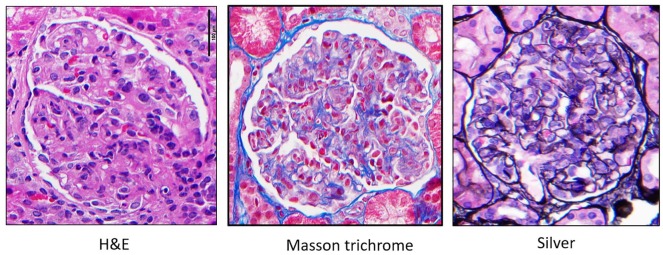
Glomeruli with global endocapillary hypercellularity with leukocytes (mainly monocytes/macrophages) obliterating most capillary lumens with different stains.

**Figure 4.**
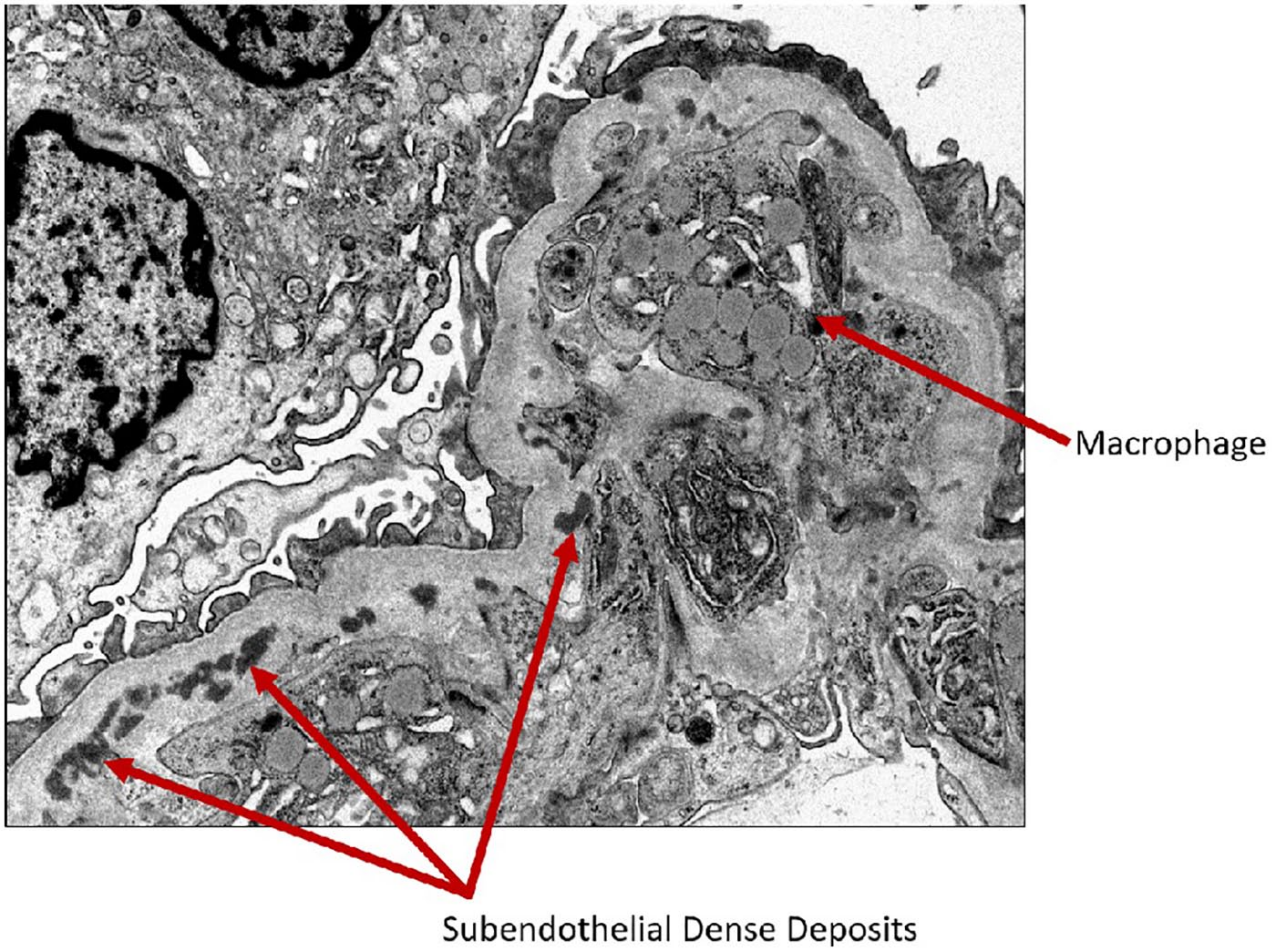
Electron microscopy showing macrophage in the capillary lumen and subendothelial dense deposits.

**Figure 5.**
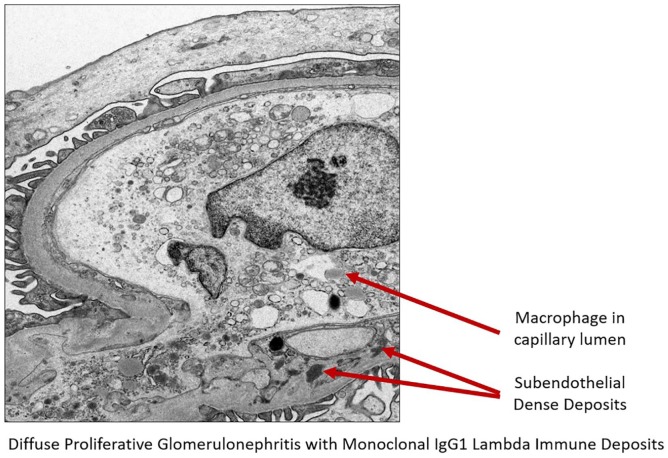
Electron microscopy showing macrophage and subendothelial dense deposits.

**Figure 6.**
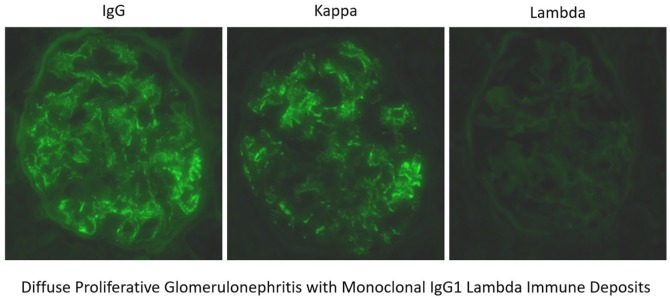
Immunofluorescence showing monoclonal immunoglobulin G1 lambda immune deposits.

She was initially treated with intravenous hydration, but her creatinine continued to escalate. Her hospital course was complicated with swelling of lips and face along with episodes of supraventricular tachycardia. She subsequently underwent a kidney biopsy because of her unusual presentation and hematuria. Kidney biopsy came positive for diffuse proliferate glomerulonephritis with monoclonal immunoglobulin G1 lambda immune deposits (Figures 1-5). An immunofluorescent study suggested a lambda light chain disease (Figure 6). Given the patient had a clinical presentation of acute angioedema along with low C1q concentration, immunoglobulin studies were checked to investigate the possibility of acquired angioedema secondary to a B-cell neoplasm. Capillary electrophoresis showed the only hypoalbuminemia with no abnormal M spike. No abnormal bands were detected on serum or urine electrophoresis. Free kappa light chain was high at 45.9 mg/dL (reference range = 3.3-19.4), free lambda light chain was high at 28.9 mg/dL (reference range = 5.7- 26.3), but the kappa-lambda light ratio was normal at 1.59. A total 24-hour urine protein was normal at 173 mg/24 h, but there was no Bence Jones proteinuria present.

She was referred to the hematologist for investigation of a possible B-cell malignancy. Hematologist did not recommend bone marrow biopsy because of unremarkable urinary electrophoresis and normal free light chain ratio. There was no clinical evidence of organomegaly or lymphadenopathy. Computed tomography scan of the abdomen did not reveal any splenomegaly or lymphadenopathy. They did not suspect she had any underlying lymphoproliferative disorder.

She was started on pulse methylprednisolone 500 mg intravenous daily and then switched to oral prednisone 60 mg daily. She had supraventricular tachycardia on day 4 of pulse steroids and hence was switched to oral prednisone. Her renal parameters improved dramatically, and her swelling and rashes subsided. She developed cellulitis of her face, and thus her steroids were discontinued after 1 week only. She was referred to a tertiary center allergy clinic who did not recommend any further specialized treatment as hereditary angioedema was not implied.

She was followed-up subsequently in the renal clinic. She had normal kidney function, but her hematuria is persistent. She is in clinical remission now with no rashes or further leg swelling. She had another episode of similar flare after 4 months and was treated with pulse steroids again. She is being followed by an allergy clinic and is carrying epinephrine pen as prophylaxis.

## Discussion

Paraproteinemias are characterized by clonal proliferation of B-cells and or plasma cells resulting in overproduction of monoclonal proteins and can cause significant renal dysfunction. Paraprotein-induced renal disease can occur without malignancy, now termed as monoclonal gammopathy of renal significance. The various mechanisms of renal injury are through paraprotein deposition or precipitation or crystallization with complement and cytokine activation.^8^ MGRS has objective evidence of renal involvement indicating end-organ damage, which differentiates from commonly known disease state MGUS. MGRS includes a wide spectrum of disorders like light and heavy chain deposition disease, C3 glomerulopathy, immunotactoid glomerulopathy, PGNMID, and primary amyloidosis.^2^

Our patient had an initial clinical presentation with urticaria, acute kidney injury, and a low complement level, raising the suspicion of acquired angioedema, possibly secondary to a monoclonal gammopathy. In a patient of this age, with the absence of family history, we abated the possibility of hereditary angioedema as a cause of her initial features. Our suspicion was validated by low C1q, which is low in acquired angioedema and normal in the hereditary form. Acquired angioedema generally presents with head and neck symptoms, mainly swollen upper airways, cheeks, and tongue.^6^ Localized, nonpruritic, subcutaneous rash, along with recurrent swellings that appear rapidly and resolve within 24 to 48 hours, is classic of this disease. Systemic manifestations are more common with the acquired angioedema and are mostly absent in hereditary angioedema. In a middle-aged or elderly patient presenting with a clinical history of angioedema, the lymphoproliferative disorder should be ruled out. In a series by Markovic et al, there was a considerable delay in the diagnosis of acquired angioedema by 2.3 years.^6^ This diagnosis has implications for multiple specialties, including head and neck surgeon, the dermatologist, the hematologist, the anesthetist, and the general practitioner. The anesthetist should be vigilant about the possibility of upper airway obstruction caused by laryngeal angioedema in this condition.^9^ Similarly, primary care physicians should be aware of this condition, given angiotensin-converting enzyme inhibitors are contraindicated.

Nasr et al in 2004 described PGNMID, a novel category of monoclonal IgG deposition disease characterized by membranoproliferative glomerulonephritis or endocapillary glomerulonephritis on light microscopy, staining for a single light chain isotype and a single gamma heavy chain subclass on immunofluorescence, and granular electron-dense deposits on electron microscopy.^10^

Our patient was diagnosed with PGNMID, which is always confirmed by kidney biopsy. Our patient also had negative serum protein electrophoresis, normal ratio of serum-free light chains, and negative urine protein electrophoresis that typically occurs in PGNMID as reported in 75% of the published cases.^11^

No treatment is indicated in MGUS as it is a benign condition, and close monitoring is warranted. The finding of MGRS on kidney biopsy necessitates more strict monitoring and necessary interventions to prevent ongoing deposition and end-organ damage.^11^ In the most extensive case series by Nasr et al with PGNMID involving 37 patients, only 38% experienced complete or partial recovery of renal function.^4^

Only one extensive review of literature by Castelli et al has shown in 35% of cases an association of MGUS and acquired angioedema.^12^ However, no recent literature has shown an association of MGRS, especially the more active PGNMID variant of the disease with acquired angioedema and low complement levels.

## Conclusion

In this report, we try to highlight this unique presentation of acquired angioedema and MGRS. Both are relatively rare but active disorders and need careful monitoring and frequent follow-ups. Further case report series needs to be done to find any large-scale association of MGRS with acquired angioedema.
